# Effect of Hydration Forms and Polymer Grades on Theophylline Controlled-Release Tablet: An Assessment and Evaluation

**DOI:** 10.3390/ph17030271

**Published:** 2024-02-21

**Authors:** Molham Sakkal, Mosab Arafat, Priya Yuvaraju, Rami Beiram, Labeeb Ali, Mohammednoor Altarawneh, Abdul Razack Hajamohideen, Salahdein AbuRuz

**Affiliations:** 1 College of Pharmacy, Al Ain University, Al Ain P.O. Box 64141, United Arab Emirates; molham.sakkal@gmail.com (M.S.); mosab.arafat@aau.ac.ae (M.A.); 2 Department of Pharmacology and Therapeutics, College of Medicine and Health Sciences, United Arab Emirates University, Al Ain P.O. Box 17666, United Arab Emirates; 3 Department of Chemical and Petroleum Engineering, United Arab Emirates University, Sheikh Khalifa Bin Zayed Street, Al Ain P.O. Box 17666, United Arab Emirates; 4 Department of Physics, United Arab Emirates University, Al Ain P.O. Box 17666, United Arab Emirates; 5 Department of Biopharmaceutics and Clinical Pharmacy, School of Pharmacy, The University of Jordan, Amman 11942, Jordan

**Keywords:** controlled-release medications, drug hydrous form, in vitro drug release, polymer grade, X-ray diffraction, scanning electron microscopy, differential scanning calorimetry, thermogravimetric analysis

## Abstract

Background: Drug release from controlled release delivery systems is influenced by various factors, including the polymer’s grade and the drug’s hydration form. This study aimed to investigate the impact of these factors on the controlled release of theophylline (THN). This research compares the monohydrate form found in branded products with the anhydrous form in generic equivalents, each formulated with different polymer grades. Methods: Quality control assessment was conducted alongside in vitro evaluation, complemented by various analytical techniques such as X-ray diffraction (XRD) and scanning electron microscopy (SEM). Additionally, thermal analyses using differential scanning calorimetry (DSC) and thermogravimetric analysis (TGA) were employed. Results: Quality control assessments demonstrated that the generic tablets exhibited lower average weight and resistance force compared to the branded ones. In vitro tests revealed that generic tablets released contents within 120 min, compared to 720 min for the branded counterpart. Characterization using XRD and SEM identified disparities in crystallinity and particle distribution between the three samples. Additionally, the thermal analysis indicated consistent endothermic peaks across all samples, albeit with minor variations in heat flow and decomposition temperatures between the two products. Conclusions: This study demonstrated that variations in polymer grade and hydration form significantly impact THN release.

## 1. Introduction

The emergence of controlled-release drug delivery systems (CDDSs) indicates a significant advancement in the pharmaceutical sector, offering controlled and pre-determined medication release [1,2]. These systems are particularly beneficial for drugs like codeine [3], hydromorphone [4], and morphine [5] as they reduce the frequency of dosing, thereby improving patient adherence, reducing side effects, and enhancing drug efficacy [6]. CDDSs are vital for drugs with a narrow therapeutic index, such as theophylline (THN) [7], and lithium [8], ensuring the maintenance of therapeutic levels and optimal patient outcomes [4,9,10]. Therefore, the development of high-quality controlled-release (CR) medications is crucial in optimizing treatment efficacy and minimizing adverse effects [11,12].

Despite the aforementioned benefits of CDDS, concerns have been raised about the quality of some CR generic medications [13,14]. This has led to several recalls, including orphenadrine citrate CR tablets [13], propoxyphene CR capsules [14], fentanyl HCL CR tablets [15], and dronabinol CR capsules [16]. The reasons behind these recalls remain ambiguous for some medications. Therefore, conducting a comprehensive investigation into the underlying causes is necessary to offer pharmaceutical companies valuable recommendations. This research aimed to thoroughly investigate the reasons behind the impaired functionality of one of the generic THN products [4,17].

**Figure 1:**
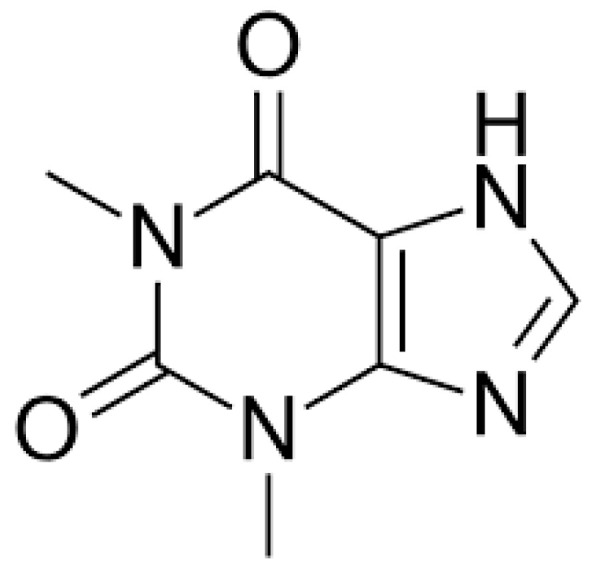
Chemical structure of theophylline [23].

Several recent studies have indicated new applications for THN, including post-tuberculous lung disease [18] and COVID-19 [7]. However, THN has a well-established history of use in treating asthma [17] and chronic obstructive pulmonary disease [19,20]. THN, also known as 1,3-dimethylxanthine (Figure 1), is a white, odorless, and crystalline powder with a molecular weight of 180.17 g/mol and a melting point of 270–274 °C [7]. The anhydrous form of THN displays a solubility of 8.75 mg/mL, while the monohydrate one exhibits a solubility of 2.99 mg/mL. Moreover, THN has a pKa value of 8.77 and a logP value of −0.02. According to the Biopharmaceutical Classification System (BCS), THN is classified as a Class I compound, characterized by its high drug solubility and permeability [21,22]. The CR dosage form is recommended for THN due to its rapid absorption, narrow therapeutic window (5 to 20 mg/mL), and short half-life (3–8 h) [20,22].

Overall, the quality of the medication is influenced by several factors: the physicochemical properties of the active ingredient, the types and proportions of excipients used, and their grades. These properties significantly impact the final product’s quality, as evidenced by various studies [24,25,26,27]. For instance, one study found that the hydrate form of cefdinir released faster than its monohydrate form [24]. Another study observed different dissolution profiles in CR matrix tablets containing acetaminophen and ibuprofen, despite similar excipients, and this was attributed to differences in the HLB values of the drugs [25].

The polymer grade is a crucial factor in the quality control and drug release profiles of pharmaceutical formulations [27,28,29,30,31]. Hartzke et al. demonstrated this in their development of 3D-printed CR tablets using various grades of hydroxyethyl cellulose (HEC), specifically 90 kDa, 300 kDa, 720 kDa, and 1000 kDa, showing significant variations in dissolution release profiles [28]. Similarly, research on different grades of polyethylene glycol (PEG)—PEG 4000, 6000, and 20,000—revealed an influence on the mechanical properties of formulations due to melting point differences [30]. Another study investigating hydroxypropyl methylcellulose (HPMC) of various grades highlighted notable differences in mucoadhesive properties linked to the viscosity of these HPMC grades [29]. These studies collectively emphasize the complexity of addressing factors that affect the functionality of recalled controlled drug release systems.

The integration of thermal and analytical techniques, complemented by in vitro assessments, forms a comprehensive approach for identifying compromised quality and functionality in CDDSs [26,32,33,34,35,36]. Essential techniques include differential scanning calorimetry (DSC), thermogravimetric analysis (TGA), X-ray diffraction (XRD), and scanning electron microscopy (SEM). For example, in vitro assessments have shown that some hydroxypropyl methylcellulose (HPMC) grades have a limited ability to sustain drug release, likely due to their lower viscosity [35]. Jannin et al. employed XRD to investigate crystallinity differences in formulations with the same drug but varying polymer grades [32]. In another study, DSC was crucial in assessing polymer compatibility with the active ingredient in CR matrix tablets for verapamil HCl, revealing substantial variations due to different polymer grades [33]. Additionally, the impact of impurities on CR medication quality was evident in a study where DSC analysis detected variations in peak temperatures and shapes, suggesting the presence of impurity [34].

This study aimed to assess the effect of varying hydration states of THN and polymer grades on drug release control in generic THN. Pure THN served as a baseline for identification, with the branded product as a reference. Hence, the monohydrate form of THN found in branded products contrasts with the anhydrous form found in generic equivalents. This research addresses the gap in understanding how different polymer grades influence drug release and formulation quality, highlighting that each of these products contains different polymer grades. A comprehensive methodology was employed, encompassing tablet quality control assessments and in vitro evaluations in both acidic pH media and distilled water. Sample characterization involved a range of thermal and analytical techniques, including DSC, TGA, XRD, and SEM.

## 2. Results

### 2.1. Quality Control Assessment

The quality control assessment of generic and branded THN products, summarized in Table 1, revealed significant differences (*p* < 0.05) in average weight, resistance force, and tablet diameter. The generic THN tablets had a lower average weight (394.43 ± 5.5 mg) compared to the branded product (414.2 ± 4.76 mg). In terms of resistance force, the branded THN showed a higher mean value (492 ± 2.83 N) compared to the generic version (120.83 ± 7.78 N). Nevertheless, both products conformed to USP standards for tablet friability, exhibiting less than 1% weight loss [37], and were within the acceptable USP weight variation limit of 7.5%. The generic product showed a marginally higher weight variation (0.19%) compared to the branded version.

### 2.2. Calibration Curve

**Figure 2:**
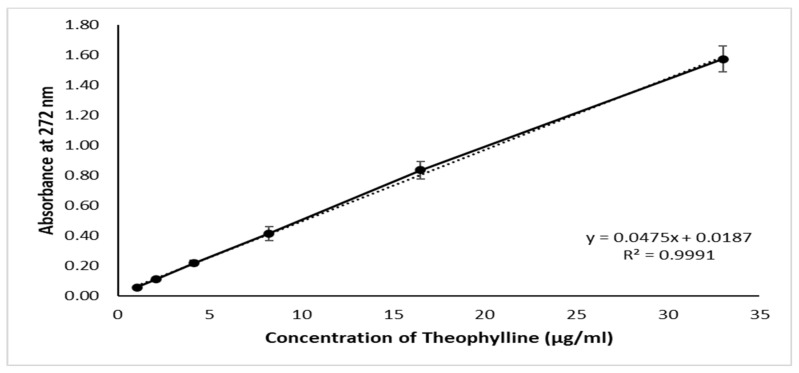
Calibration curve for theophylline detection by UV–visible spectrophotometry at 272 nm (average ± standard deviation, sample size = 3).

Figure 2 presents a calibration curve that is notably precise, evidenced by its correlation coefficient (*r*^2^) of 0.9991. This demonstrates the curve’s robustness and reliability in accurately determining drug concentrations in unknown samples.

### 2.3. In Vitro Drug Release

**Figure 3:**
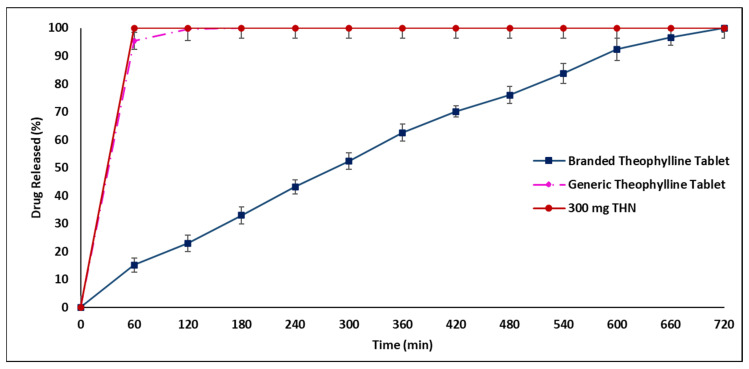
Comparative release profiles of branded and generic theophylline tablets and 300 mg THN in distilled water over 12 h at 37 °C (mean ± SD, *n* = 3).

**Figure 4:**
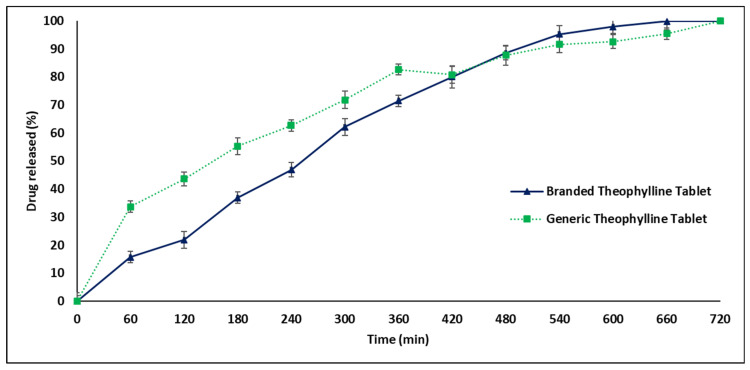
Comparative release profiles of branded and generic theophylline tablets in acidic media over 12 h at 37 °C (mean ± SD, *n* = 3).

**Figure 5:**
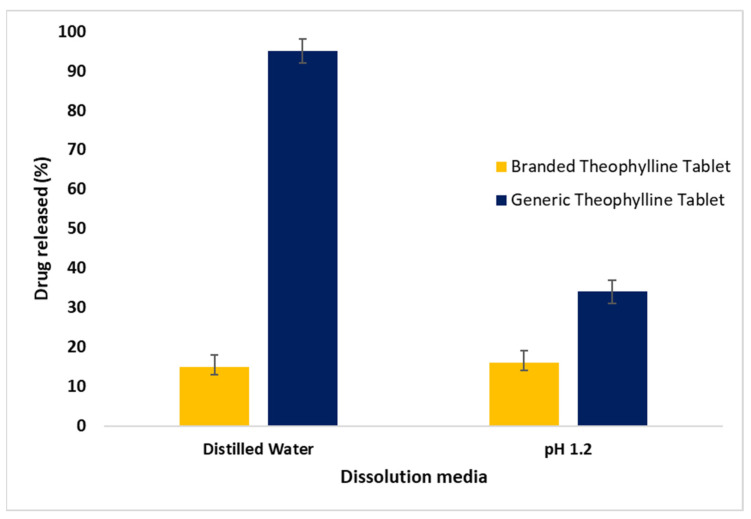
Drug release profiles at 60 min for branded and generic theophylline tablets in distilled water and acidic media (mean ± SD, *n* = 3).

Figure 3 presents a significant difference (*p* < 0.05) in the drug release profiles of generic and branded THN products in distilled water. The generic tablets released their contents prematurely within 120 min, in contrast to the CR over 720 min achieved by the branded product. This rapid release by the generic tablet deviates from FDA guidelines for CR medications, which stipulate no more than 30% drug release in the first 2 h [3]. Additionally, the branded product demonstrated a more delayed release compared to the generic THN profile. In acidic media (pH 1.2), significant differences in drug release patterns were also observed between the products, with the branded one exhibiting more effective control (*p* < 0.05). As shown in Figure 4, nearly 50% of the drug was released from the generic product after 120 min, compared to about 20% from the branded product. Figure 5 further illustrates that the release rate of the generic product varied with the media, releasing approximately 35% in acidic conditions and 95% in distilled water within the first hour, whereas the branded product maintained a consistent release rate in both media types after one hour.

### 2.4. Characterization Using Thermal and Analytical Techniques

#### 2.4.1. X-ray Diffraction

**Figure 6:**
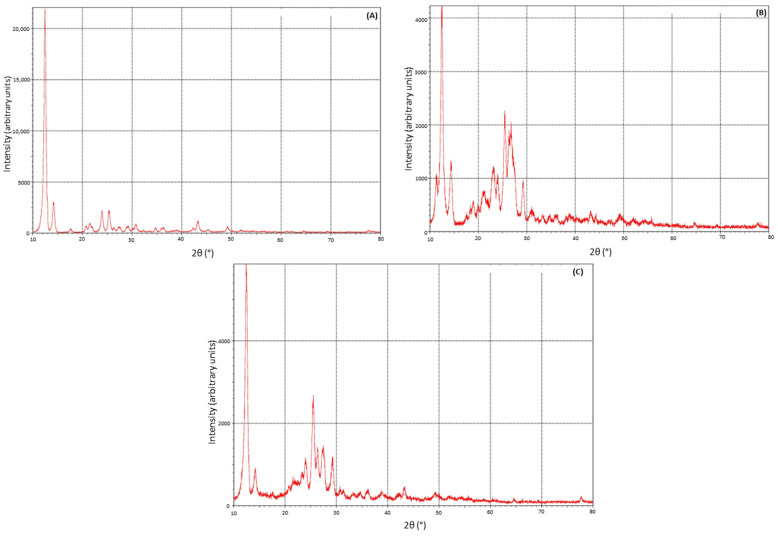
Comparative X-ray diffraction analysis of (**A**) pure theophylline powder, (**B**) branded product of theophylline, and (**C**) generic product of theophylline.

The XRD analysis results, depicted in Figure 6, showed the diffraction patterns of THN, and both the branded and generic THN products. THN drug exhibited distinct, sharp peaks, particularly a prominent peak near a 2θ value of 12°, along with other peaks at 14.3°, 24.1°, and 25.5°, consistent with its crystalline structure. This corresponds with the established literature on THN characteristics. The branded and generic products also exhibited these primary peaks, yet they showed varying intensities. The order of peak intensity, from highest to lowest, was observed as follows: pure THN powder, the generic product, and finally the branded product. Additionally, the XRD patterns of both the branded and generic products revealed unique peaks that were not present in the pure THN powder.

#### 2.4.2. Scanning Electron Microscopy

**Figure 7:**
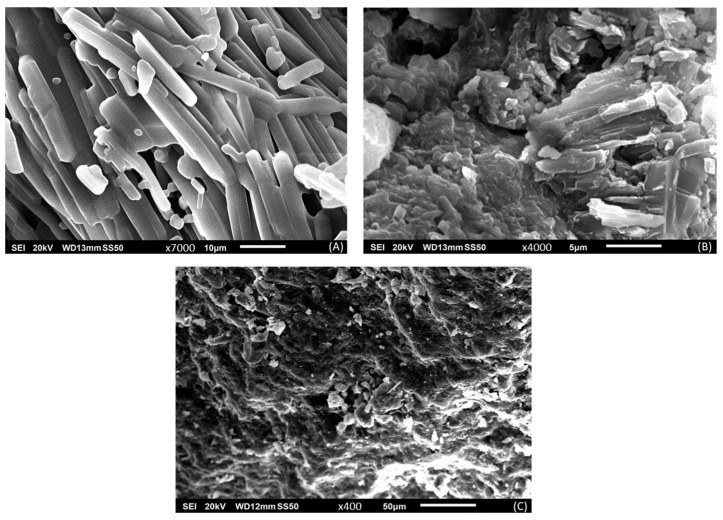
Comparative scanning electron microscopy analysis of (**A**) pure theophylline powder, (**B**) branded product of theophylline, and (**C**) generic product of theophylline.

Figure 7 presents the SEM results for pure THN powder, as well as for the branded and generic products of THN. The pure THN powder exhibits a regular shape with well-defined edges; the particles are uniformly arranged with dimensions mostly less than 10 µm in width and greater than 10 µm in length, presenting rod-like structures. In contrast, SEM images of both the generic and branded products reveal irregular particle shapes and distribution.

#### 2.4.3. Thermal Analysis

**Figure 8:**
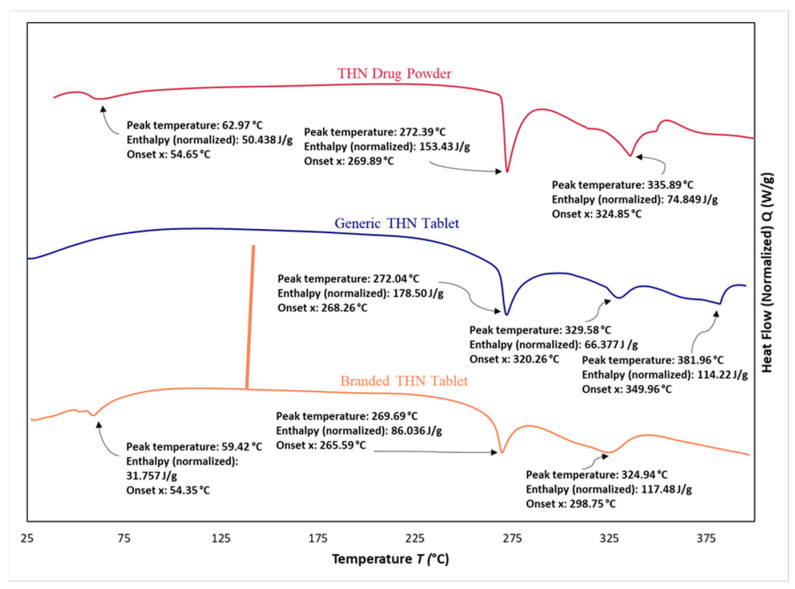
Comparative analysis using differential scanning of THN drug powder, generic THN tablet, and branded THN tablet (*n* = 3).

Figure 8 demonstrated the thermal profiles obtained using DSC for the pure powder of THN, the branded product of THN, and the generic product of THN. In the DSC thermogram, a consistent endothermic peak was observed across all three samples. Conversely, the pure powder of THN exhibited an endothermic peak at 272.39 °C, characterized by a (*T*_onset_) of 269.89 °C, and an enthalpy of 153.43 J/g. In comparison, the branded THN product displayed a similar endothermic peak at 269.69 °C, with a heat absorption value of 86.036 J/g, and a (*T*_onset_) of 265.59 °C. Similarly, the generic THN product showed an endothermic peak at 272.04 °C, accompanied by a heat absorption of 178.50 J/g, and a (*T*_onset_) at 268.26 °C. The heat absorption was the lowest for the branded sample. The endothermic peak, representing the melting points of the three samples, showed no significant difference (*p* > 0.05). However, a significant difference in heat absorption was observed between the branded THN product and the other two THN samples (*p* < 0.05).

Additionally, a less intense endothermic peak was detected exclusively in the pure powder and the branded THN product. This peak occurred at 62.97 °C for the pure THN powder and at 59.42 °C for the branded product. Moreover, at 141.28 °C, an exothermic peak was observed in the DSC thermogram of both the pure and branded THN, a feature absents in the thermogram of the generic sample.

**Figure 9:**
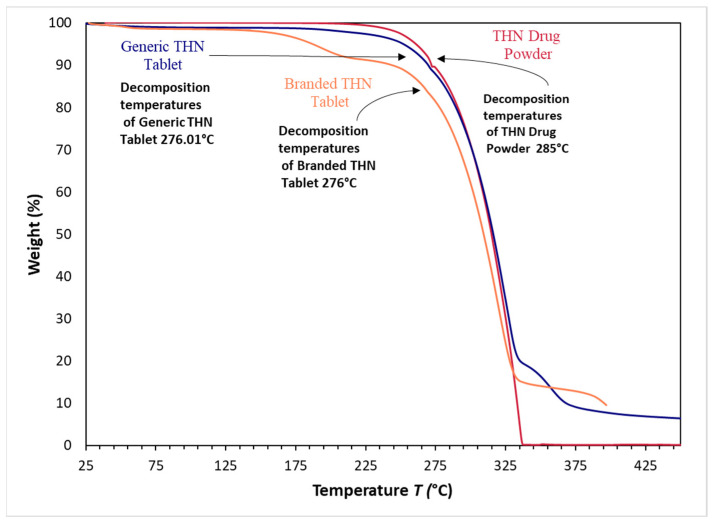
Comparative analysis using thermogravimetric analysis of THN drug powder, generic THN tablet, and branded THN tablet (*n* = 3).

In terms of TGA, the results indicated nearly identical decomposition profiles for all three samples, as shown in Figure 9. The decomposition temperatures were recorded as 285 °C for the pure THN powder, 276 °C for the branded THN product, and 276.01 °C for the generic THN product. Although no significant difference (*p* > 0.05) was observed between the branded and generic products, a significant difference (*p* < 0.05) was noted between them and the pure powder. Additionally, there was a slight variation in the temperature required to achieve a 50% weight loss in the samples: approximately 315 °C for both the THN powder and the generic tablet, compared to 309 °C for the branded tablet. The main thermal events obtained using DSC and TGA for the three THN samples are presented in Table 2.

## 3. Discussion

This research provides comprehensive insights into the effects of various polymer grades on CDDS and their effect on the drug release profile of THN. This study also delves into the impact of different hydration states of THN on its release. Notably, it is among the first studies to extensively evaluate the influence of specific polymers, such as polyvinylpyrrolidone (PVP), PEG, and HEC, on the premature release of THN. Furthermore, this study delves into a relatively less explored domain, investigating the influence of varying hydration states of the active pharmaceutical ingredient on drug release dynamics. These findings highlight the importance of selecting suitable polymer grades in pharmaceutical manufacturing.

In the tablet quality assessment, the observed variation in average weight between the branded and generic THN tablets indicates potential differences in the types, ratios, and grades of excipients used in each [38]. The slightly lower weight variation in the branded THN product may suggest a more consistent manufacturing process and superior powder flowability compared to the generic versions [39,40].

The observed differences in mean resistance force between the branded and generic THN tablets could be attributed to the use of distinct PVP polymer grades. Specifically, PVP K-90 was utilized in the branded tablets, while PVP K-30 was used in the generic versions. A higher molecular weight of PVP K-90 contributes to stronger binding and enhanced cohesive properties, which in turn may lead to improved mechanical strength and hardness in the branded tablets. This aligns with findings from previous studies, where PVP K90 was shown to offer superior binding properties in paracetamol tablets compared to PVP K30 [41]. This insight into the impact of PVP polymer grades on tablet properties recommends that additional examinations be performed, such as in vitro dissolution studies.

The significant observed variations in in vitro drug release profiles between generic and branded tablets in distilled water might be attributed to several factors. A primary consideration is the use of different hydration forms of THN in the products—monohydrate in the branded and anhydrous in the generic versions—which is presumed to influence drug release patterns [42,43].

The monohydrate form of THN in the branded product is assumed to provide greater stability, primarily due to stronger intermolecular bonds formed by additional hydrogen bonding with water molecules. This can lead to reduced solubility in dissolution fluids [42,43,44]. The integration of a water molecule into the drug’s structure might also limit its exposure to the dissolution media, consequently slowing down the dissolution process [43,45]. Additionally, hydrate forms generally exhibit lower solubility than their anhydrous counterparts due to decreased Gibbs free energy [43]. These findings align with previous studies indicating that the anhydrous form of THN has higher solubility compared to the monohydrate form, which significantly affects drug release [42,44].

The variations in drug release profiles between the products might also be attributed to the use of different polymer grades. The branded product incorporated HEC 300 kDa and PEG 8 KDa, whereas the generic product used HEC 30 kDa and PEG 6 KDa. It is assumed that the differences in molecular weight of these polymer grades are a primary factor affecting the drug release variation. Polymers with higher molecular weights form larger molecular chains, leading to a denser and more extended network in drug formulations. This dense network acts as a robust barrier to drug diffusion, resulting in a slower drug release rate from the dosage form. Moreover, the higher molecular weight of the polymer correlates with increased gel viscosity upon contact with dissolution media, further retarding drug release over an extended period [28,46,47].

The results of our study are consistent with existing research. For instance, previous research has shown that using PEG 8000 instead of PEG 6000 in azithromycin formulations leads to an extended period of controlled drug release [46]. Additionally, another study indicated that ibuprofen tablets containing HEC with a molecular weight of 300 KDa exhibited prolonged drug release compared to those with HEC 90 KDa [28]. These findings highlight the significant role of polymer molecular weight in affecting drug release rates.

The dissolution behavior of the generic and branded THN products in acidic media revealed distinct release profiles. The generic product displayed a pH-dependent release, whereas the branded product showed a pH-independent release pattern. This variation is assumed to be attributed to the differing solubility of the two hydrous forms of THN. Specifically, the anhydrous THN in the generic product is more soluble in water than in acidic media, in contrast to the monohydrate THN in the branded product, which exhibits only slight solubility differences in both media. These findings align with previous research on rifampicin, where solubility variations were noted between its monohydrate and anhydrate forms in water and acidic environments [48].

The variation observed between the two products in XRD peak intensity is presumed to result from differences in the hydration forms of the drug molecules in the samples, as corroborated by previous studies, including one by Liu et al. [49]. Additionally, the appearance of new peaks in the XRD spectra of both products could be due to the inclusion of polymeric excipients in the formulations. Notably, variations in these peaks among the products suggest the use of different polymer grades [33]. This is further supported by SEM analysis, which demonstrated significant irregularities in the structure of both products [50].

The slight differences in melting points observed through DSC for the two products imply that both tablets contain the same drug molecule. Specifically, the melting points of these samples are in agreement with those identified for pure THN powder. This observation aligns with previous research findings: Devi et al. [51] and Otsuka et al. [52] reported melting points for THN at 272.6 °C and 269.7 °C, respectively.

Additionally, the observed decrease in heat flow in the branded sample could be attributed to reduced crystallinity in both the excipients and the drug, likely resulting from the efficient dispersion of the drug molecule within the carrier system. The enhancement in dispersion may be due to variations in the polymer grade. This observation is consistent with the understanding that changes in polymer characteristics can significantly affect the physical properties of drug formulations [53].

Our results align with a study conducted by Bouriche et al., in which a polylactic acid microparticle loaded with metformin was formulated. The DSC analysis of this formulation revealed a significant decrease in the heat flow, interpreted as a sign of effective drug dispersion within the polymer matrix [11]. Additionally, the higher crystallinity of the generic tablet, as indicated by TGA, is evidenced by the slightly higher temperature required to achieve a 50% weight loss compared to the branded tablet.

The presence of a distinctive endothermic peak within the 59 to 63 °C range in both the pure THN powder and the branded product is assumed to be associated with the evaporation of water molecules, considering that both the pure powder and the branded product are monohydrates. On the other hand, the absence of this peak in the generic counterpart supports the assumption that the generic product uses an anhydrous form of THN [54].

## 4. Material and Methods

### 4.1. Materials

The pure THN powder was secured from Sigma Aldrich (Merck, St. Louis, MO, USA), while both the generic and branded versions of THN were acquired from a pharmaceutical distributor.

### 4.2. Quality Control Assessments

#### 4.2.1. Weight Variation Analysis of Samples

To evaluate the weight variation for the generic product of THN and the branded product of THN, a sensitive digital scale (Shimadzu, Kyoto, Japan) was utilized. For each product, the weight of 20 tablets was measured and compared to the average weight. This approach enabled the determination of whether each tablet’s weight fell within the acceptable standard deviation of 7.5%, as stipulated by USP regulations to ensure consistency and high quality in medication [55].

#### 4.2.2. Tablet Friability Testing

The friability test was conducted to assess the durability of tablets for the generic product of THN and the branded one. This test was carried out using a friabilator TA 220 testing apparatus (Erweka GmbH, Heusenstamm, Germany), which rotates the tablets in a drum at 25 revolutions per 1 min for 4 min. Following the test, the tablets were reweighed, and the percentage of weight loss was calculated. This percentage was then compared to the maximum allowable weight loss limit of 1% specified by USP regulations [56]. A total of 20 tablets from each THN product were tested, and the results were recorded.

#### 4.2.3. Assessment of Tablet Crushing Strength

The mean resistance force of the tablets for the two products was determined using a TBH-225 TD hardness tester (Erweka GmbH, Heusenstamm, Germany). A total of 20 tablets were randomly selected from each of the two THN products, and their mean resistance force was measured. The maximum force required to fracture the tablet was recorded in newtons (N).

### 4.3. Calibration Curve

A THN-branded tablet was finely crushed using a mortar and pestle. A base solution of 0.33 mg/mL was created by dissolving this powder in 100 mL of distilled water and stirring at 250 RPM for an hour, with subsequent additions of water up to a total of 900 mL to ensure complete dissolution. The solution was then diluted to obtain concentrations of 33, 16.5, 8.25, 4.12, 2.06, and 1.03125 µg/mL. UV spectrophotometry measured the absorbance for each concentration, yielding values of 1.57, 0.83, 0.41, 0.22, 0.11, and 0.06, which were plotted on a calibration curve against the concentrations. A high regression factor of 0.9991 indicated excellent linearity. This process was performed three times to confirm accuracy, with the average results documented.

### 4.4. In Vitro Dissolution Test

To evaluate the drug release of the THN products, a Dis 8000 dissolution apparatus (Copley Scientific, Nottingham, UK) was used with stirring speed set to 100 rpm. Following USP guidelines, the test solution comprised 0.1 N hydrochloric acid with pH adjusted to 1.2, totaling a volume of 900 mL. Additionally, distilled water was used as another incubation medium. The temperature of the incubation media was consistently maintained at 37 ± 0.5 °C throughout the experiment.

At specified intervals (60, 120, 180, 240, 300, 360, 420, 480, 540, 600, 660, and 720 min), 5 mL samples were drawn from each vessel and immediately replaced with an equal volume of the corresponding incubation media. These samples were then diluted with distilled water and filtered to eliminate any particulate matter. The filtered samples were analyzed using ultraviolet spectrophotometry at a wavelength of 271 nm to determine the drug concentration. This study was carried out with three samples of each product (*n* = 3).

The calibration curve allowed for the accurate measurement of sample concentrations, which was used to analyze the release profiles of the generic and branded versions of THN. This was done by plotting the concentration on the x-axis and the corresponding absorbance on the y-axis.

### 4.5. Characterization Using Thermal and Analytical Techniques

#### 4.5.1. X-ray Diffraction

XRD analysis was performed using an XRD 6100 system (Shimadzu, Kyoto, Japan) to determine the crystalline structures of the THN samples. The instrument scanned the 2θ angle range from 10° to 80° at a rate of 0.02 degrees per min. The obtained diffraction patterns were analyzed with specialized software to identify and compare the characteristic peaks of each product.

#### 4.5.2. Scanning Electron Microscopy

SEM analysis was conducted using a JSM-6010PLUS/LA SEM (JEOL Ltd., Tokyo, Japan) in order to investigate the microstructural characteristics of the THN samples. A small amount of the powder adhered to the sample holder using double-sided adhesive tape and then coated with a thin layer of gold using a Cressington sputter coater, enhancing contrast and minimizing charging effects. The imaging was performed under high vacuum conditions with an acceleration voltage of 20 kV.

#### 4.5.3. Differential Scanning Calorimeter

DSC was conducted using a DSC-60 Plus instrument (Shimadzu, Kyoto, Japan). The three samples were analyzed with 3–5 mg of powder from each product after it had been transferred into sample pans for analysis. The experiment was carried out under controlled conditions, and we scanned the samples over a temperature range of 25–350 °C at a rate of 10 °C per min under a continuous nitrogen flow of 100 mL/min. Data were collected and analyzed using Lab Solutions TA 60 software. This study was carried out with three samples of each product (*n* = 3).

#### 4.5.4. Thermogravimetric Analysis

TGA was conducted to measure the thermal stability of the three samples using a TGA-50 instrument (Shimadzu, Kyoto, Japan). A precise amount of 10–15 mg of each sample was weighed and transferred into sample pans. The analysis was performed under controlled conditions, where the samples were heated over a temperature range of 0 °C to 600 °C at a rate of 15 °C per min in a nitrogen environment with a constant flow rate of 50 mL/min. The resulting data were collected and processed using the Lab Solutions TA software. This study was carried out with three samples of each product (*n* = 3).

### 4.6. Statistical Analysis

For statistical analysis, the one-way ANOVA was utilized to compare the mean values of the measured variables. A significance threshold of *p* < 0.05 was established to identify significant differences. This analysis was performed using the Statistical Package for the Social Sciences (SPSS) Version 26.

## 5. Conclusions

In conclusion, this study has provided substantial evidence that variations in polymer grades have a significant impact on the properties and functionality of CDDS. These variations notably affect key parameters related to quality control, in vitro drug release profiles, crystallization tendencies, and thermal behaviors. This study has highlighted the pivotal role of polymer grade variations in influencing the drug release functionality of generic tablets and highlighted the importance of selecting the appropriate drug hydration form during formulation development. Further research is needed to enhance our understanding of the combined impact of different polymer grades and hydrate forms on the efficacy of CDDS.

## Figures and Tables

**Table 1:** Quality control assessment for the generic product of THN and branded product of THN.

Quality Assessment Parameter	The Generic Product of THN	The Branded Product of THN
Average weight (mg)	394.43 ± 5.5 *	414.2 ± 4.76
Weight variation range (%)	0.95 ± 0.93	0.76 ± 0.80
Tablet friability (%)	0.17 ± 0.02	0.16 ± 0.001
Mean resistance force (N)	120.83 ± 7.78 *	492 ± 2.83
Mean tablet diameter (mm)	12.03 ± 0.01 *	6.02 ± 0.01

* Significant difference (*p* < 0.05).

**Table 2:** Differential Scanning Calorimetry and Thermogravimetric Analysis Thermal Properties of THN Drug Powder, Generic THN Tablet, and Branded THN Tablet.

Sample	Melting Temperature (°C)	Enthalpy of Melting (J/g)	Onset Temperature (°C)	Maximum Decomposition Temperature (°C)
THN Drug Powder	272.39	153.43	269.89	285.00 *
Generic THN Tablet	272.04	178.50	268.26	276.01
Branded THN Tablet	269.69	86.036 *	265.59	276.00

* Significant difference (*p* < 0.05).

## Data Availability

Data is contained within the article.
